# Characteristics of *Pinus hwangshanensis* Rhizospheric Fungal Community along Huangshan Mountain’s Elevation Gradients, China

**DOI:** 10.3390/jof10100673

**Published:** 2024-09-27

**Authors:** Qinglin Zuo, Keke Dang, Jing Yin, Dandan Yuan, Jing Lu, Xingjia Xiang

**Affiliations:** 1 School of Resources and Environmental Engineering, Anhui University, Hefei 230601, China; qinglinzuo@163.com (Q.Z.); yinjing01102023@163.com (J.Y.); lujing22163@163.com (J.L.); 2 Anhui Province Key Laboratory of Wetland Ecosystem Protection and Restoration, Hefei 230601, China; 3 International Collaborative Research Center for Huangshan Biodiversity and Tibetan Macaque Behavioral Ecology, Hefei 230601, China

**Keywords:** elevation gradient, rhizospheric fungi, community composition, assembly process, plant pathogen

## Abstract

Elevation gradients strongly influence the diversity pattern of soil microorganisms. To date, many studies have elucidated the response of soil microbes to changes in elevation gradients. However, the effects of these gradients on the assembly mechanisms and network complexity of rhizospheric microbial communities remain underexplored. To bridge this knowledge gap, this study assessed the response of rhizospheric fungal communities of *Pinus hwangshanensis* along different elevation gradients in the Huangshan Mountain scenic area with regard to diversity, community composition, and assembly mechanisms using high-throughput amplicon sequencing. The results revealed significant differences in rhizospheric fungal community composition across three elevation gradients. The soil organic matter and pH were the most relevant factors influencing the changes in rhizospheric fungal community composition. The rhizospheric fungal diversity was significantly lower at both low and high elevations compared to the medium elevation. The rhizospheric fungal community assembly showed a more deterministic process at low and high elevations than at the medium elevation, indicating that stronger environmental filtering contributed to reduced fungal diversity at the extremes of the elevation gradient. In addition, rhizospheric pathogens, particularly Dermateaceae, acted as keystone taxa, diminishing the stability of co-occurrence networks at the medium elevation. This study contributes to a more comprehensive understanding of rhizospheric fungal community patterns and their ecological functions along elevation gradients in mountainous regions.

## 1. Introduction

As an important component of terrestrial ecosystems, mountains are critical for maintaining biodiversity and ecosystem functions [1]. Microorganisms significantly contribute to mountain ecosystems, and they are closely related to soil functional diversity and nutrient cycling within these environments [2,3]. Soil microbial communities are spatially dynamic and complex, exhibiting different structures and functions in the soil at different elevations [4]. In addition, environmental heterogeneity induced by elevation affects microbial turnover, which has indirect effects on ecosystem function [5,6]. A number of environmental factors, such as soil total nitrogen (TN) and pH, have been shown to vary with elevation gradients, thereby influencing microbial community composition [7]. These variations in soil properties shape the microbial distribution pattern along elevation gradients. Therefore, investigating the effects of elevation gradients on soil fungal community distribution may enhance our understanding of the mechanisms underlying microbial spatial pattern formation and maintenance.

To date, numerous studies have elucidated the responses of soil microbial communities to changes along elevation gradients. However, the impact of elevation gradients on the characteristics of rhizospheric microbial communities remains largely unexplored. The plant rhizosphere is regarded as one of the most active and complex ecosystems, enhancing plants’ ability to withstand both abiotic and biotic pressures [8]. Rhizospheric microorganisms are important drivers that directly or indirectly influence the ecological health of the plant rhizosphere, and the robustness of microbial communities colonizing the plant rhizosphere contributes to nutrient uptake for their hosts and promotes plant growth [9]. Microorganisms also play integral roles in soil remediation by breaking down some of the harmful organic matter [10,11]. In addition, rhizospheric microorganisms promote plant survival under unfavorable conditions by enhancing the plant’s ability to resist attack by pathogens in the soil [12]. However, the fungal community’s ecological function and diversity in the rhizosphere are strongly impacted by multiple environmental factors [13]. Therefore, the study of the rhizosphere’s microbial composition is a very important guide to promoting plant health and resilience in mountainous regions.

Mountain soil microorganisms undergo ecological succession influenced by the interplay of environmental dispersal and environmental filtration due to the geological conditions of mountain ranges [14]. A related study found that the assembly process has different effects on microbial communities at different elevations [15]. Currently, with the continuous development of high-throughput sequencing technology, an improved understanding of fungal diversity, community composition, and potential functions has been gained at different elevations. The well-preserved ecosystem of Huangshan Mountain, with noticeable vertical geographic differences, serves as a natural laboratory for studying the elevation distribution pattern of soil microbial communities [16]. As one of the “three wonders” of Huangshan Mountains, *Pinus hwangshanensis* is the most common and important tree species in the Huangshan Mountain area, and it is also an endemic conifer species in China, possessing considerable economic and ecological value [17]. Despite the recognized importance of rhizospheric fungi for plants, knowledge regarding the distribution of rhizospheric fungal communities along elevation gradients remains limited.

To understand the ecological functions of the rhizospheric fungal community of *P. hwangshanensis*, this study used high-throughput amplicon sequencing technology to analyze the fungal spatial distribution, symbiotic networks, assembly mechanisms, and effects of environmental factors on the rhizospheric fungal communities at three different elevations (800 m, 1000 m, and 1200 m) in Huangshan Mountain. The following two hypotheses were tested: (1) elevation gradients lead to differences in the diversity and structure of rhizospheric fungal communities; (2) the assembly processes and potential functions of rhizospheric fungal communities vary across elevation gradients.

## 2. Materials and Methods

### 2.1. Site Selection and Soil Sampling

The study area was located on Huangshan Mountain (118°01′–118°14′ E, 30°01′–30°18′ N), and sample collection took place in September 2022. Firstly, we selected three elevation gradients: 800 ± 20 masl, 1000 ± 20 masl, and 1200 ± 20 masl. At each elevation, eight adult *Pinus hwangshanensis* trees were selected with an inter-distance of more than 20 m. For each tree, a sample was collected by taking five random rhizospheric soil samples tightly attached to the roots in the root system of *P. hwangshanensis* and mixing them into one sample, with eight soil samples at each elevation [18]. The rhizospheric soil samples were preserved on ice. After removing prominent leaves and dead branches, the soil samples were divided into two portions. One portion was stored at 4 °C for soil chemical analysis, and the other was stored at −80 °C for DNA extraction.

### 2.2. Determination of Soil Chemical Properties

The suspension was shaken well at a soil–water mass ratio of 1:2.5 (*v*/*v*) and left to stand for 30 min, after which the soil pH was measured using a pH meter. Soil moisture (SM) was determined by drying. The soil organic matter (SOM) and total nitrogen (TN) contents were determined using an elemental analyzer (EA3000, Euro Vector, Pavia, Italy). The total phosphorus (TP), available phosphorus (AP), and alkaline nitrogen (AN) were determined using standard methods [19,20,21].

### 2.3. DNA Extraction and PCR Amplification

Soil DNA was extracted from 24 separate soil samples using the FastDNA Spin Kit (MP Biomedicals, Santa Ana, CA, USA)for Soil according to the manufacturer’s instructions. The extracted DNA was identified using 1% agarose gel electrophoresis and quantified with ultraviolet spectrophotometry (Eppendorf, Germany) [22]. The ITS1 region of the fungal ITS gene was amplified using the ITS1F (5′-CTTGGTCATTTAGAGGAAGTAA-3′) and ITS2R (5′-GCTGCGTTCTTCATCGATGC-3′) primers to obtain fungal data [23]. The PCR products were extracted from 2% agarose gel and then purified using the AxyPrep DNA Gel Extraction Kit (Axygen Biosciences, Union City, CA, USA).

### 2.4. Sequence Data Processing

We used QIIME1 (v1.9) for the quality control (average quality score > 30) of raw data from the MiSeq platform and USEARCH (version 8.0.1623) to perform operational taxonomic unit (OTU) assignment using >97% as the shared identity threshold [24]. The most abundant sequence within each OTU was selected as a representative sequence identified by the ribosomal database project Classifier with the UNITE (7.2) database. The randomly selected subsets of 61,000 sequences per sample were used for further analysis (Figure S1). The raw data have been submitted to the NCBI (National Center for Biotechnology Information) under project number SRP507372.

### 2.5. Statistical Analysis

A one-way ANOVA (analysis of variance) was performed to compare the differences between groups at the *p* < 0.05 level in SPSS 20.0. The differences in community composition were characterized using the non-metric multidimensional scaling analysis (NMDS) method. The effect of environmental variables on fungal community composition was analyzed using Mantel’s test, the variance inflation factor (VIF), and redundancy analysis (RDA) using the R (4.4.1) package vegan (2.6-4). The effects of nesting and turnover on fungal community structure were investigated based on the decomposition of β-diversity by Jaccard variability in the adespatial package (0.3-23) in the R (4.4.1) software [25]. The network parameters were calculated through the R packages “psych” (2.4.3) and “igraph” (2.0.3) and finally visualized with Gephi (0.9.2). Neutral community modeling predicted the relationship between the frequency of OTU occurrence and its relative abundance in fungal communities from different elevations [26]. The β-null model was used to distinguish fungal community assembly processes [27]. Rhizospheric fungal potential functions were predicted using FUNGuild analysis [28].

## 3. Results

### 3.1. Soil Chemical Properties

Different elevation gradients had significant effects on the rhizospheric soil properties of *Pinus hwangshanensis*. Soil pH gradually decreased along the elevation gradients (Table 1). TN, AN, and TP were significantly higher at a high elevation than at low and medium elevations (*p* < 0.05; Table 1). AP and SOM showed an increasing trend along the elevation gradients (Table 1).

### 3.2. Fungal Alpha Diversity

**Figure 1:**
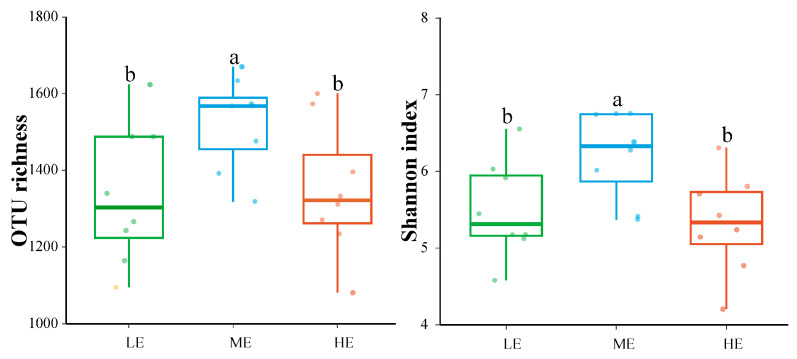
Rhizospheric fungal richness and Shannon index of *Pinus hwangshanensis* at different elevations. Letters represent significant differences from the one-way ANOVA with Duncan’s comparisons (*p* < 0.05).

A total of 2,965,667 filtered fungal sequences and 8521 fungal OTUs were detected in all soil samples. There were 348 fungal OTUs (10.65%) shared in the rhizosphere of *P. hwangshanensis* among the three different elevations (Figure S2). The unique fungal OTUs were 2407, 2608, and 2424 for low, medium, and high elevations, respectively (Figure S2). The rhizospheric fungal α-diversity (OTU richness and Shannon index) showed a unimodal pattern with the highest values measured at mid-elevation compared to low and high elevations (*p* < 0.05) (Figure 1).

### 3.3. Fungal Community Composition

**Figure 2:**
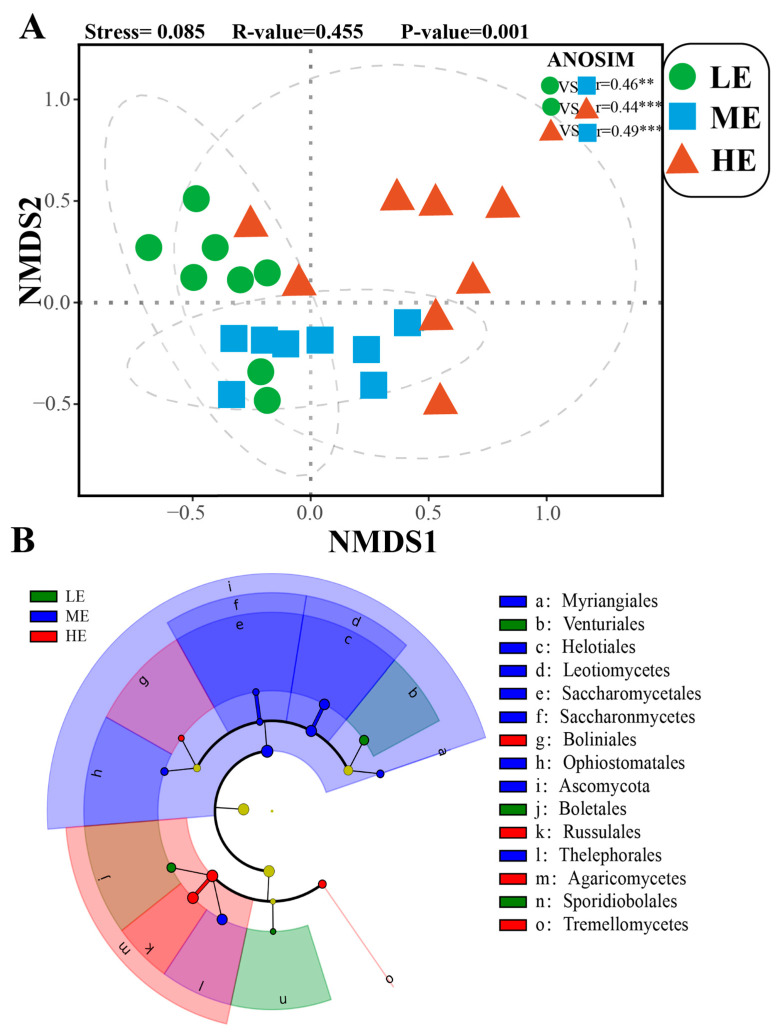
(**A**) NMDS of fungal community composition based on Bray–Curtis distance and differences in community composition between different elevations tested using ANOSIM, ** *p* < 0.01, *** *p* < 0.01. (**B**) The LEfSe analyses show rhizospheric soil fungal biomarkers associated with each elevation (effect sizes greater than 2 and α values less than 0.05). LE: low elevation; ME: medium elevation; and HE: high elevation.

The predominant fungal phyla in the rhizosphere were Ascomycota (75.27%), Basidiomycota (20.21%), Zygomycota (4.37%), and Chytridiomycota (0.15%) (Figure S3). The relative abundance of Ascomycota was higher in the mid-elevation soil samples compared to the other samples. The relative abundance of Basidiomycota was higher in the high-elevation soil samples (Figure S3). The low-elevation soil samples had the highest relative abundance of Zygomycota and the lowest relative abundance of Chytridiomycota (Figure S4). Fungal community composition (β-diversity) showed significantly different values between the elevation points (Figure 2A). The LEfSe results showed that fungi were enriched in three orders (Venturiales, Boletales, and Sporidiobolales) in the low-elevation rhizospheric soil samples; the higher fungal relative abundance was found in one phylum (Ascomycota), two classes (Leotiomycetes and Saccharomycetes), and five orders (Myriangiales, Helatiales, Saccharomycetales, Ophiostomatales, and Thelephorales) in the medium-elevation rhizospheric soil samples; two classes (Agaricomycetes and Tremellomycetes) and two orders (Boliniales and Russulales) showed enrichment in high-elevation rhizospheric soil samples (Figure 2B). The indicator analysis showed that there were three (*Russula*, *Elaphomyces,* and *Clitocybula*), seven (*Oidiodendron*, *Hypomyces*, *Umbelopsis*, *Tomentella*, *Lactarius*, *Lachnum*, and *Hymenoscyphus*), and six (*Hypocrea*, *Mortierella*, *Russula, Hypomyces, Cadophora,* and *Hymenoscyphus*) enriched fungal genera at low, medium, and high elevations, respectively (Table 2).

**Figure 3:**
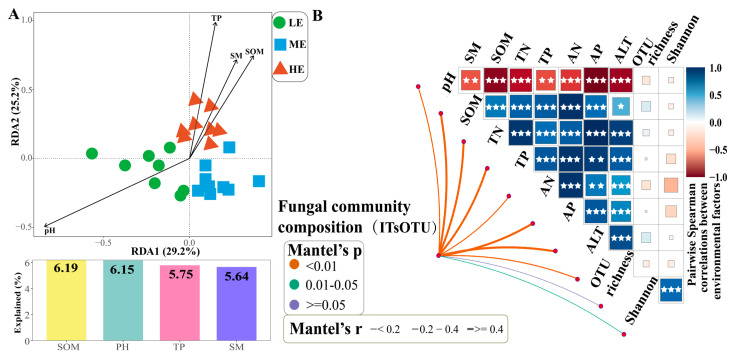
(**A**) Redundancy analysis (RDA) showing the effects of major environmental factors on rhizospheric fungal communities at different elevations. (**B**) Spearman’s correlation analysis was used for correlation analyses between major environmental factors and indices of fungal community diversity (OTU richness and Shannon index), and Mantel’s test was used to test for correlation between the composition of fungal communities and environmental factors. The size of the square corresponds to the value of the correlation coefficient, the width of the line corresponds to the Mantel distance correlation r statistic, and the color of the line indicates the Mantel *p*-value, * *p* < 0.05, ** *p* < 0.01, *** *p* < 0.001.

**Figure 4:**
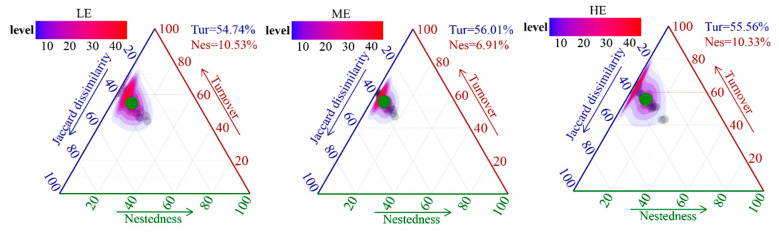
Triangulation of β-diversity patterns based on Jaccard differences in fungal community composition at different elevations. Each point is determined by the values in the Jaccard dissimilarity, nestedness, and turnover matrices.

The results of RDA combined with the variance partitioning analysis (VPA) showed that the differences in fungal community structure in rhizospheric soils at different elevations were mainly influenced by SOM (6.19%), pH (6.15%), TP (5.75%), and SM (5.64%) (Figure 3A). Mantel’s test showed that fungal community composition had the highest correlation with AN (r = 0.501, *p* < 0.001), TN (r = 0.494, *p* < 0.001), and SOM (r = 0.477, *p* < 0.001) (Figure 3B). Furthermore, the dominant fungal genera (*Umbelopsis*, *Chloridium*, *Cryptococcus,* and *Alatospora*) were strongly associated with these soil properties according to the correlation analysis (Figure S5). To uncover possible intrinsic mechanisms that maintain the structural stability of fungal communities at different elevations, this study further decomposed the structural differences among the communities into turnover and nested components. Overall, taxonomically based differences in community structure were dominated by species turnover (>50%), whereas the contribution of nesting was relatively lower, especially at the medium elevation (6.91%) (Figure 4).

### 3.4. Potential Functional Fungi in Rhizospheric Soil

**Figure 5:**
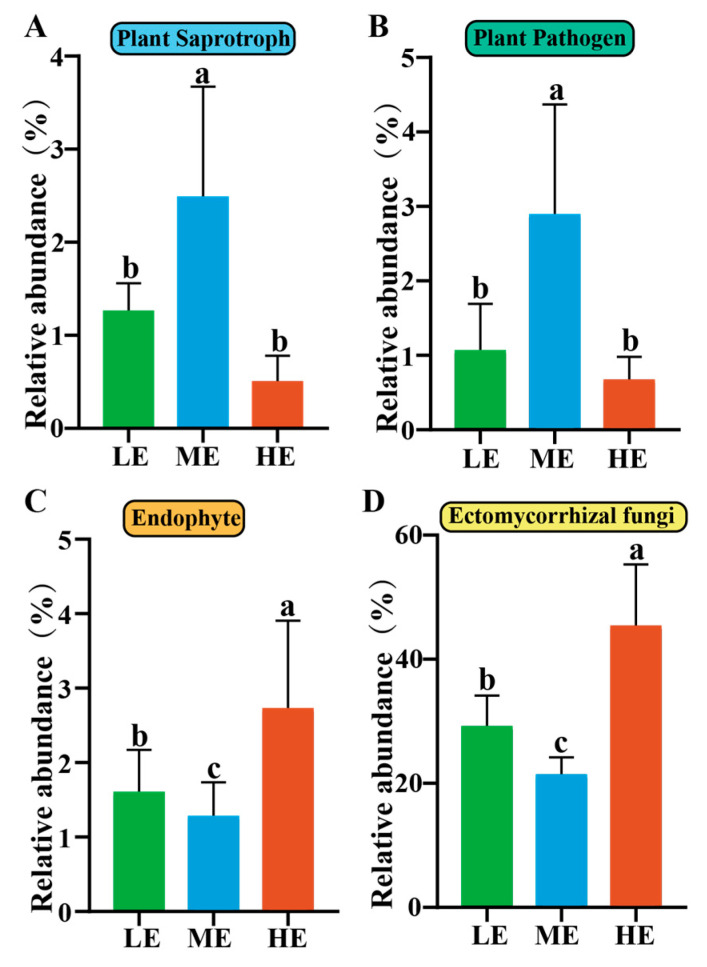
The fungal predicted function. (**A**) Potential plant saprophytes in the rhizosphere at different elevations. (**B**) Potential plant pathogen in the rhizosphere at different elevations. (**C**) Potential endophyte in the rhizosphere at different elevations. (**D**) Potential ectomycorrhizal fungi in the rhizosphere at different elevations. Letters represent significant differences derived from the one-way ANOVA with Duncan’s comparisons (*p* < 0.05).

The FUNGuild function prediction analysis provided valuable insights into the functional roles of different groups of fungi present in the rhizospheric soil of *P. hwangshanensis* forests. The results revealed the presence of plant saprotrophs, plant pathogens, endophytes, and ectomycorrhizal fungi in the collected samples, with variations between different elevations. Specifically, the analysis identified 2 plant saprotrophic OTUs, 10 plant pathogenic OTUs, 15 endophyte OTUs, and 77 ectomycorrhizal OTUs across the samples. The relative abundance of plant saprotrophs and pathogens was higher at a medium elevation (Figure 5A,B). In contrast, endophytes and ectomycorrhizal fungi were more abundant at higher elevations (Figure 5C,D).

### 3.5. Community Assembly Mechanisms of Rhizospheric Fungi

**Figure 6:**
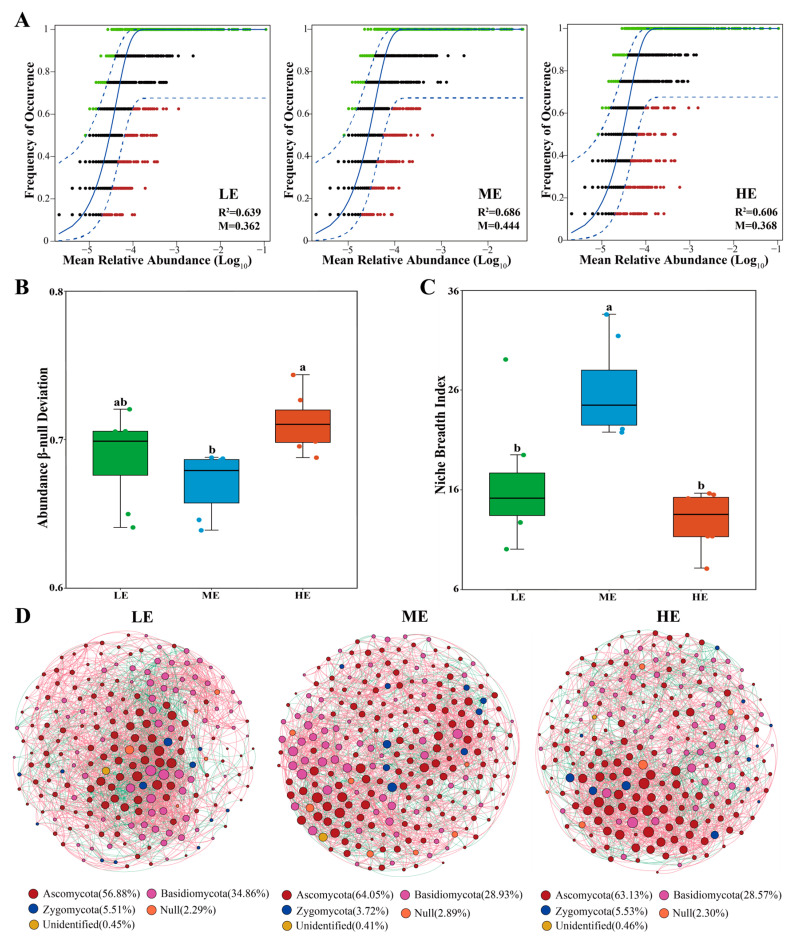
(**A**) Neutral community model. OTUs that occur more frequently than the model-predicted values are shown in green, OTUs that occur less frequently than the predicted values are shown in red, and OTUs that occur within the predicted range are shown in black. The dashed line indicates the 95% confidence interval around the model-predicted value (blue line). R^2^ represents the overall goodness of fit of the neutral community model. The M-value quantifies the migration rate at the community level, with a smaller M-value indicating a more restricted dispersal of species throughout the community and a higher M-value indicating a less restricted dispersal. (**B**) The abundance-based β-null model distinguishes the relative importance of deterministic and stochastic processes by the value of zero deviation. the closer the value of β-diversity deviation is to zero, the greater the stochasticity; the closer the value of β-diversity deviation is to one, the greater the determinism. (**C**) Niche width was used to show the utilization of resources by fungi in each elevation. Letters represent significant differences from the one-way ANOVA with Duncan’s comparisons (*p* < 0.05). (**D**) The co-occurrence network structure of rhizospheric fungal community with OTU’s relative abundance >0.01% at the phylum level among the three elevations. Null is an unrecognized phylum of fungi. LE: low elevation; ME: medium elevation; and HE: high elevation.

The mobility of fungi in the soil was estimated using the neutral model, which revealed that the mobility of rhizospheric fungi was greater in the mid-elevation soil samples (M = 0.44) than in the low-elevation (M = 0.36) and high-elevation soil samples (M = 0.37), showing that the fungal community was more restricted to dispersal in low- and high-elevation soils than in mid-elevation soil (Figure 6A). The β-null deviation values in the fungal communities differed significantly across elevations, with lower values observed in the mid-elevation group, showing that stochastic processes drove fungal community assembly in mid-elevation soil, while deterministic processes mainly shaped fungal community assembly in low- and high-elevation soils (Figure 6B). Niche width is an important measure to evaluate microbial environmental adaptation. Based on the calculated niche width results, the niche width was wider for mid-elevation than for low- and high-elevation rhizospheric fungal communities (Figure 6C). In addition, the main environmental variables were selected to determine the correlation between the assembly process of fungal communities and environmental factors. The abundance β-null deviation values were significantly and positively correlated mainly with SOM (r = 0.335, *p* < 0.05) and TP (r = 0.340, *p <* 0.05) (Figure S6).

### 3.6. Analysis of Rhizospheric Fungal Networks

The co-occurrence networks of rhizospheric fungi at different elevations were established using Spearman’s correlation analysis. The network complexity was highest at low elevations as the network had the highest edges, average density, and clustering coefficients and the shortest average path lengths compared to medium and high elevation (Table S1). Network stability, assessed by natural connectivity, was also highest at low elevations and lowest at a medium elevation (Figure S7). Ascomycota was the predominant phylum in the rhizospheric fungal network across the three elevations (Figure 6D). Keystone species play important roles in determining the function and interactions of fungal communities, and species with a high degree (>19) and low betweenness centrality (<150) values in the fungal network are considered to be keystone species. The key species found in this study at each elevation are listed in Table S2.

## 4. Discussion

Understanding the effect of elevation on microbial community patterns and soil chemical properties is important for clarifying fungal-driven ecosystem functioning [29]. Soil nutrient contents, especially SOM, increased significantly along the elevation gradients, possibly because a lower temperature triggered the slow growth of plants and the accumulation of nutrients at higher elevations (Table 1). However, the soil pH showed a decrease along the elevation gradients, as the accumulation of organic matter might have increased the content of humic acid [30]. Environmental heterogeneity strongly influenced the spatial distribution of fungal communities [31,32], consistent with the findings of the present study (Figure 3A). Different soil environments along the elevation gradients created a certain niche, affecting fungal community composition [33,34]. In this study, the SOM content showed a significant increase along the elevation gradients. The SOM, as an important source of nutrients for fungal growth, was closely related to the fungal community composition [35]. Changes in the SOM content might drive a shift in fungal community composition along elevation gradients.

Differences in the spatial turnover rates of fungal communities, driven by dispersal constraints and niche selection, resulted in alterations in the spatial distribution patterns of fungal community composition at different elevations [36]. The contribution of nesting was higher at both low and high elevations relative to the medium elevation (Figure 4). Higher nesting promoted biotic homogenization, leading to a loss of alpha diversity [37,38], which may be an important factor contributing to the lower fungal diversity at low and high elevations (Figure 1). Furthermore, the neutral model and null model analyses suggested that stochastic processes were the dominant mechanism driving the assembly process of soil fungal communities at the medium elevation, whereas deterministic processes dominated the soil fungal communities at the low and high elevations (Figure 6B,C), further demonstrating that higher environmental filtering decreases soil fungal diversity at low and high elevations. Moreover, the soil samples collected at low and high elevations showed a lower fungal niche width than the soil samples collected at a medium elevation (Figure 6C). Previous studies have shown that niche width decreases as the environment becomes hostile, and inter-competition increases among taxa [39], thereby decreasing microbial diversity.

The predicted fungal functions were investigated in this study. The medium-elevation soil had higher relative abundance for plant saprotrophs group. The relative abundance of Ascomycota was highest at the medium elevation (Figure S4). Previous studies have shown the prominent role of Ascomycota in decomposing lignocellulose [40], which is consistent with the predicted function result. The greater abundance of plant saprotroph indicated a strong cycling of organic matter, which might have been induced by the greater fungal species turnover mentioned above at the medium elevation. However, the relative abundance of plant pathogens was higher at the medium elevation. A greater abundance of pathogens might be unfavorable to the growth of *Pinus hwangshanensis*. The soil samples collected at a high elevation had the highest relative abundance of endophyte and ectomycorrhizal fungi. Both endophyte and ectomycorrhizal fungi are important plant-beneficial fungi (PBF). Endophyte and ectomycorrhizal fungi have proven to be extremely important plant companions that can increase the resilience of the host and promote plant survival in soil [41,42]. As the environmental conditions at high elevations are harsher, *P. hwangshanensis* might rely more on their rhizospheric PBF to growth.

Fungal co-occurrence network complexity and stability are closely related to topological parameters, including nodes, edges, clustering coefficients, average variation degree (AVD), and natural connectivity [43,44]. The stability of the fungal networks at the mid-elevation was lower than that of the networks at the low and high elevations (Figure S7 and Table S1). Keystone taxa are essential for maintaining network function and structure. For instance, the Trichocomaceae family is likely to play a significant role in soil aggregation through its adhesive properties, and some species in the genus *Oidiodendron* have been shown to secrete growth hormones and enzymes that have a variety of beneficial effects on host plants (Table S2) [45,46,47,48]. The functional diversity provided by these low-elevation network species may be crucial for enhancing the overall stability of an ecological network. In this study, one of the keystone taxa (Dermateaceae) in the mid-elevation network was found to be a plant pathogen that causes the necrosis of plant leaves (Table S2) [49,50]. It has been demonstrated that pathogens decrease the stability of networks [51,52]. In this study, the mid-elevation rhizospheric soil was invaded by a large number of fungal plant pathogens (Figure 5B). Thus, the lower stability of the mid-elevation network might have been due to the presence of antagonism between pathogens and other fungal taxa. When the stability of fungal symbiotic networks is low, it may reduce the stability and sustainability of soil functions [53]. Therefore, there is a potential risk of pathogen outbreaks in mid-elevation environments that may impact plant health and ecosystem function, and attention should be paid to preventing the spread of plant pathogens in the rhizospheric soil of *P. hwangshanensis*.

## 5. Conclusions

In conclusion, our findings highlight significant differences in the fungal community structures within the rhizospheric soils of *Pinus hwangshanensis* across varying elevation gradients. The observed changes in soil properties likely drive the distinct fungal community patterns associated with these gradients. Notably, we found that the lower fungal diversity at both low and high elevations is attributed to a higher degree of fungal homogenization and more intense environmental filtering compared to mid-elevation areas. At high elevations, the relative abundance of putative endophytic and ectomycorrhizal fungi was markedly higher, indicating that *P. hwangshanensis* may increasingly depend on these beneficial fungi to withstand the challenging environmental conditions found in such habitats. Conversely, the mid-elevation zone exhibited a greater abundance of rhizospheric plant pathogens, which could pose a threat to the growth and health of *P. hwangshanensis*. This study enriches our understanding of the complex dynamics of rhizospheric fungal communities and their ecological functions within mountain ecosystems along elevation gradients. By elucidating these relationships, our research not only contributes to the broader field of microbial ecology but also has important implications for the conservation and management of *P. hwangshanensis* forests in the face of environmental changes.

## Figures and Tables

**Table 1:** Soil chemical properties at different elevations.

Soil Properties	SM(%)	pH	TN(g/kg)	AN (mg/kg)	AP (mg/kg)	TP (g/kg)	SOM (g/kg)
LE	32.54 ± 9.95 b	4.87 ± 0.19 a	2.60 ± 0.77 b	331.51 ± 105.55 b	3.28 ± 0.79 c	0.18 ± 0.06 b	109.20 ± 14.06 c
ME	39.67 ± 14.61 b	4.44 ± 0.14 b	3.51 ± 1.03 b	380.31 ± 157.79 b	11.63 ± 4.02 b	0.16 ± 0.05 b	153.85 ± 24.58 b
HE	56.81 ± 17.13 a	4.18 ± 0.08 c	7.47 ± 1.68 a	700.86 ± 118.30 a	24.38 ± 10.67 a	0.41 ± 0.07 a	281.93 ± 64.47 a

The values in brackets represent the standard deviation of the mean. Letters represent significant differences from the one-way ANOVA with Duncan’s comparisons (*p* < 0.05).

**Table 2:** Indicator analyses showing genera with a relative abundance greater than 0.1% at each elevation. LE: low elevation; ME: medium elevation; and HE: high elevation.

Treatment	Indicator Value	*p*	Taxonomy	Relative Abundance (%)
LE	1.771	0.026	*g__Russula*	2.193
	0.975	0.03	*g__Elaphomyces*	0.278
	0.830	0.005	*g__Clitocybula*	0.247
ME	2.675	0.055	*g__Oidiodendron*	0.582
	0.921	0.029	*g__Hypomyces*	0.910
	1.582	0.002	*g__Umbelopsis*	0.639
	1.659	0.006	*g__Tomentella*	0.474
	0.838	0.023	*g__Lactarius*	0.389
	0.912	0.001	*g__Lachnum*	0.301
	0.828	0.013	*g__Hymenoscyphus*	0.293
HE	1.618	0.004	*g__Hypocrea*	2.221
	0.679	0.009	*g__Mortierella*	1.145
	0.933	0.029	*g__Russula*	0.616
	0.887	0.013	*g__Hypomyces*	0.509
	0.855	0.005	*g__Cadophora*	0.211
	0.894	0.027	*g__Hymenoscyphus*	0.206

## Data Availability

The raw data were submitted to the NCBI (National Center for Biotechnology Information) under project number SRP507372.

## Supplementary Materials

The following supporting information can be downloaded at https://www.mdpi.com/article/10.3390/jof10100673/s1, Table S1: Co-occurrence network topological features statistics; Table S2: Keystone species in co-occurrence network; Figure S1: Rarefaction curves of the number of operational taxonomic units (OTUs) at 97% similarity boxplot for each of 24 samples; Figure S2: Venn diagrams showing endemic and shared fungal OTUs in three different elevation rhizosphere; Figure S3: The relative abundance of rhizosphere fungal phyla at each sampling site; Figure S4: The differences of dominant rhizosphere fungal phyla at different elevations; Figure S5: Spearman rank correlations between the relative abundance of dominant fungal taxa and soil properties; Figure S6: Linear regression analysis of soil physicochemical and β-null deviation; Figure S7: The stability of co-occurrence network. Reference [54] is cited in the Supplementary Materials.
