# Supplementary material for: Characteristics of Pinus hwangshanensis Rhizospheric Fungal Community along Huangshan Mountain’s Elevation Gradients, China
Source: J Fungi (Basel). 2024 Sep 27;10(10):673. doi: 10.3390/jof10100673 (PMC11508824; doi:10.3390/jof10100673)
Supplement: Supplementary file 1 [file jof-10-00673-s001.zip › jof-3181034-supplementary.pdf]

Supporting Information

Supporting Materials and Methods

The extracted DNA was diluted to 20 ng/μL and used as a template for PCR. All soil DNA concentrations ranged from 1.8-2.0. DNA diluent was mixed with Phusion® high-fidelity, PCR master mix (New England Biolabs Inc., Ipswich, MA, USA). The PCR amplification procedure is described as follows: first, the samples were heated to 98°C for 30 s, denatured at 98°C for 10 s, annealed at 55°C for 30 s, extended at 72°C for 1 min, followed by 25 cycles of 98°C for 10 s, and stored at 72°C for 10 min [1]. The PCR amplifications of the ITS1 region was examined by agarose gel (See figure below). Second, AMPure XP beads (Beckman Coulter, Indianapolis, IN, USA) were used to purify the DNA. Finally, the purified DNA was duplex sequenced (2×250) on an Illumina MiSeq platform.

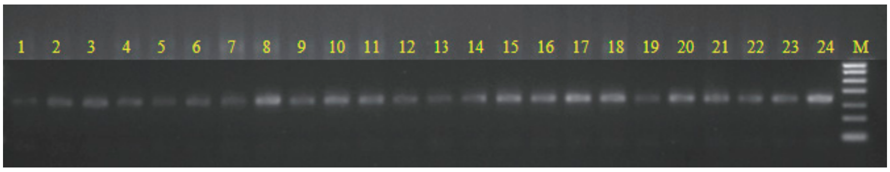

Supporting reference

Chen, Z., Zheng, Y., Ding, C., Ren, X., Yuan, J., Sun, F., Li, Y. Integrated metagenomics and molecular ecological network analysis of bacterial community composition during the phytoremediation of cadmium-contaminated soils by bioenergy crops. *Ecotoxicol. Environ. Saf.* **2017** 145, 111–118.

The main QIIME1 code:

```
1.join_paired_ends.py -f r1.fq -r r2.fq -j 10 -p 10 -o joined_pairs

2.multiple_split_libraries_fastq.py -i fq/ -o split -m sampleid_by_file --
include_input_dir_path --remove_filepath_in_name -p
/home/qiime/Desktop/QIIME_parameters.txt

3. pick_otus.py -i split/seqs.fna -m usearch --db_filepath=unite_97.fasta -o
usearch_qf_results/ --word_length 64 --non_chimeras_retention=union -g 2

3.pick_rep_set.py -i usearch_qf_results/seqs_otus.txt -f split/seqs.fna -r
unite_97.fasta -o rep_set.fna
```

4.assign_taxonomy.py -i rep_set.fna -r unite_97.fasta -t unite_97_taxonomy.txt -m rdp

5.make_otu_table.py -i usearch_qf_results/seqs_otus.txt -t rdp_assigned_taxonomy/rep_set_tax_assignments.txt -o otu_table_all.biom

6. filter_otus_from_otu_table.py -i otu_table_all.biom -o otu_table2.biom -n 2

7. biom summarize-table -i otu_table2.biom -o out_table_summary.txt

8. beta_diversity_through_plots.py -i otu_table2.biom -m map.txt -o bdiv_even61000 -e 61000

9.summarize_taxa_through_plots.py -i bdiv_even61000/otu_table2_even61000.biom -o wf_taxa_summary -m map.txt

10.multiple_rarefactions.py -i otu_table2.biom -m 10 -x 61000 -s 60990 -n 20 -o rarefied_otu_tables/

11.alpha_diversity.py -i rarefied_otu_tables/ -m observed_species,chao1,shannon,simpson_e -o alpha_div/

12.collate_alpha.py -i alpha_div/ -o collated_alpha/

13.biom convert -i bdiv_even61000/otu_table2_even61000.biom -o otu_table61000.txt --to-tsv --header-key taxonomy --table-type="OTU table"

The main R code:

1. Box chart code

```
library(ggplot2)
mydata <- read.csv("shannon.csv",header = T,row.names = 1)
windowsFonts(A=windowsFont("Times New Roman"),
        B=windowsFont("Arial"))
e <- ggplot(mydata, aes(x = dose, y = shannon ))
p <- e + geom_boxplot(aes(color = dose),width=0.65,lwd=1.4) +
  scale_x_discrete(limits=c("LE","ME","HE"))+#
  scale_fill_manual(values = c("#43AA46", "#25A0DC", "#CC522A"))+
  geom_jitter(aes(colour=dose),
```

```
        position = position_jitter(0.25),size=3,alpha=0.6)+
scale_colour_manual(values = c("#43AA46", "#25A0DC", "#CC522A"))+
theme_classic()+scale_y_continuous(limits = c(4,8.1),expand = c(0,0))+
theme(axis.ticks.length=unit(0.25, "cm"),
    axis.text.x = element_text(margin=unit(c(0.5,0.5,0.5,0.5), "cm")),
    axis.text.y = element_text(margin=unit(c(0.5,0.5,0.5,0.5), "cm")) )+
theme(text=element_text(family="A",size=20))+
theme(axis.text.x = element_text(angle = 0,hjust = 0.2,vjust = 0.2))+
theme(axis.line = element_line(color = "black", size = 1.0, linetype = "solid"))+
theme(axis.title = element_text(size = 20,colour = "black"))+
theme(axis.text = element_text(colour = "black",size = 20,angle = 0,vjust = 0.5))+
theme(legend.title =  element_text(size = 20,colour = "black"),
    legend.text = element_text(size = 20,colour = "black"),
    strip.text =  element_text(size = 20,colour = "black"),
    text = element_text(family = "serif"))
```

2. Redundancy analysis code

```
library(vegan)
library(ggplot2)
df <- read.table("111.txt",sep="\t",header = T,row.names = 1,check.names = F)
df <-data.frame(t(df))
env <- read.delim('env.txt', row.names = 1, sep = '\t', stringsAsFactors = FALSE,
       check.names = FALSE)
env <- env[-1]
env <- env[-2]
env <- env[-5]
env <- env[-6]
env <- env[-4]
env <- env[-5]
head(df)
```

```
head(env)
print(decorana(df))
df <-data.frame(t(df))
head(df)
head(env)
print(decorana(t(df)))
RDA <- rda(df,env,scale = TRUE)
RDA
vif.cca(RDA)
df_rda <- data.frame(RDA$CCA$u[,1:2],rownames(env))
df_rda
colnames(df_rda)=c("RDA1","RDA2","samples")
df_rda
df_rda_score <- data.frame(RDA$CCA$v[,1:2])
df_rda_score
RDA1 =round(RDA$CCA$eig[1]/sum(RDA$CCA$eig)*100,2)
RDA2 =round(RDA$CCA$eig[2]/sum(RDA$CCA$eig)*100,2)
group <- read.table("group.txt", sep='\t', header=T)
group
colnames(group) <- c("samples","group"
RDA.scaling1 <- summary(RDA, scaling = 1)
RDA.scaling1 RDA.perm=permutest(RDA,perm=999)
RDA.perm
RDA_env <- envfit(RDA~., data = env, perm = 999, choices = c(1,2), display =
    'sites')
RDA_env
r2 <- RsquareAdj(RDA)
rda_noadj <- r2$r.squared
rda_noadj
rda_adj <- r2$adj.r.squared
```

```
rda_adj
```

3. Mantel test code

```
library(linkET)
library(ggplot2)
library(dplyr)
micro <- read.delim('1.txt', row.names = 1)
micro <- t(micro)
micro
env <- read.delim(' 111.txt', row.names = 1)
env
correlate(env)
correlate(env) %>%
  as_md_tbl() %>%
  qcorrplot() +
  geom_square()
correlate(micro, env)
correlate(micro, env) %>%
  qcorrplot() +
  geom_square() +
  scale_fill_gradientn(colours = RColorBrewer::brewer.pal(11, "RdBu"))
mantel <- mantel_test(spec = micro, env = env, spec_select =
    list(Pathogen_community= 1:3), mantel_fun = 'mantel')
mantel<-mantel %>%
  mutate(rd = cut(r, breaks = c(-Inf, 0.2, 0.4, Inf),labels = c("< 0.2", "0.2 - 0.4",
    ">= 0.4")),
      pd = cut(p, breaks = c(-Inf, 0.01, 0.05, Inf), labels = c("< 0.01", "0.01 - 0.05",
    ">= 0.05")))
#> `mantel_test()` using 'bray' dist method for 'spec'.
```

4. Neutral model code

```
library(Hmisc)
library(minpack.lm)
library(stats4)
spp<-read.csv('otu3.txt',head=T,stringsAsFactors=F,row.names=1,sep = "\t")
spp1=spp[,1:8]
spp <- t(spp1)
N <- mean(apply(spp, 1, sum))
p.m <- apply(spp, 2, mean)
p.m <- p.m[p.m != 0]
p <- p.m/N
spp.bi <- 1*(spp>0)
freq <- apply(spp.bi, 2, mean)
freq <- freq[freq != 0]
C <- merge(p, freq, by=0)
C <- C[order(C[,2]),]
C <- as.data.frame(C)
C.0 <- C[!(apply(C, 1, function(y) any(y == 0))),]
p <- C.0[,2]
freq <- C.0[,3]
names(p) <- C.0[,1]
names(freq) <- C.0[,1]
d = 1/N
##Fit model parameter m (or Nm) using Non-linear least squares (NLS)
m.fit <- nlsLM(freq ~ pbeta(d, N*m*p, N*m*(1 -p),
    lower.tail=FALSE),start=list(m=0.1))
m.fit #get the m value
m.ci <- confint(m.fit, 'm', level=0.95)
freq.pred <- pbeta(d, N*coef(m.fit)*p, N*coef(m.fit)*(1 -p), lower.tail=FALSE)
pred.ci <- binconf(freq.pred*nrow(spp), nrow(spp), alpha=0.05, method="wilson",
```

```
        return.df=TRUE)
Rsqr <- 1 - (sum((freq - freq.pred)^2))/(sum((freq - mean(freq))^2))
Rsqr# get the R2 value
bacnlsALL <-data.frame(p,freq,freq.pred,pred.ci[,2:3])
inter.col<-rep('black',nrow(bacnlsALL))
inter.col[bacnlsALL$freq <= bacnlsALL$Lower]<-'#A52A2A'#define the color of
        below points
inter.col[bacnlsALL$freq >= bacnlsALL$Upper]<-'#4BFF00'#define the color of
        up points
library(grid)
grid.newpage()
pushViewport(viewport(h=0.6,w=0.6))
pushViewport(dataViewport(xData=range(log10(bacnlsALL$p)),
        yData=c(0,1.02),extension=c(0.02,0)))
grid.rect()
grid.points(log10(bacnlsALL$p),
        bacnlsALL$freq,pch=20,gp=gpar(col=inter.col,cex=0.7))
grid.yaxis()
grid.xaxis()
grid.lines(log10(bacnlsALL$p),bacnlsALL$freq.pred,gp=gpar(col='blue',lwd=2),
        default='native')
grid.lines(log10(bacnlsALL$p),bacnlsALL$Lower  ,gp=gpar(col='blue',lwd=2,lty
        =2),default='native')
grid.lines(log10(bacnlsALL$p),bacnlsALL$Upper,gp=gpar(col='blue',lwd=2,lty=
        2),default='native')
grid.text(y=unit(0,'npc')-unit(2.5,'lines'),label='Mean Relative Abundance
        (Log10)', gp=gpar(fontface=2))
grid.text(x=unit(0,'npc')-unit(3,'lines'),label='Frequency of
        Occurence',gp=gpar(fontface=2),rot=90)
draw.text <- function(just, i, j) {
```

```
grid.text(paste("Rsqr=",round(Rsqr,3),"\n","Nm=",round(coef(m.fit)*N)), x=x[j],
        y=y[i], just=just)
 #grid.text(deparse(substitute(just)), x=x[j], y=y[i] + unit(2, "lines"),
 #         gp=gpar(col="grey", fontsize=8))
}
x <- unit(1:4/5, "npc")
y <- unit(1:4/5, "npc")
draw.text(c("centre", "bottom"), 4, 1)
```

5. Network code

```
library(Hmisc)
library(psych)
library(reshape2)
library(igraph)
library(openxlsx)
data <- read.delim('HE.txt', row.name = 1, check.names = FALSE)
head(data)
otu <- t(data)
otu <- otu[,colSums(otu)/sum(otu)>=(0.04/100)]
write.csv(otu,file="otu.csv")
otu <- read.csv("otu.csv", header=T, row.names=1,  comment.char="")
otu <- t(otu)
data_ccor                                                          =
      corr.test(t(otu),use="pairwise",method="spearman",adjust="fdr",alpha=0.
      05)
data_ccor.r = data_ccor$r
data_ccor.p = data_ccor$p
data_ccor.r[data_ccor.p>0.05|abs(data_ccor.r)<0.8] = 0
diag(data_ccor.r) <- 0
```

Table S1: Co-occurrence network topological features statistics. LE: low elevation; ME: middle elevation; HE: high elevation.

Stages	LE	ME	HE
**Nodes**	218	242	217
**Edges**	2397	1904	1825
**Density**	0.094	0.065	0.078
**Modularity**	1.809	1.614	1.539
**Clustering coefficient**	0.46	0.41	0.44
**Path length (Average)**	2.599	2.716	2.657
**AVD**	0.67	0.68	0.66

Table S2: Keystone species in co-occurrence network. LE: low elevation; ME: middle elevation; HE: high elevation.

Stages	OTU	Degree	Betweenness centrality	Taxonomy
LE	OTU3543	41	144.55	f__ Trichocomaceae
	OTU3543	41	131.25	f__ Herpotrichiellaceae
	OTU5164	47	116.47	*g__ Oidiodendron*
	OTU1372	40	141.54	*g__ Hypocrea*
ME	OTU952	20	133.24	f__Dermateaceae
	OTU5424	24	139.77	*g__Russula*
	OTU7329	21	102.40	*g__Umbelopsis*
	OTU1685	20	143.53	*g__Acrodontium*
	OTU2809	20	101.48	*g__ Cladophialophora*
HE	OTU3048	21	118.35	f__Herpotrichiellaceae
	OTU4841	31	141.16	*g__Devriesia*
	OTU7172	30	94.37	*g__Lachnum*
	OTU7146	29	107.00	*g__Cadophora*
	OTU1085	23	67.42	*g__ Hypocrea*

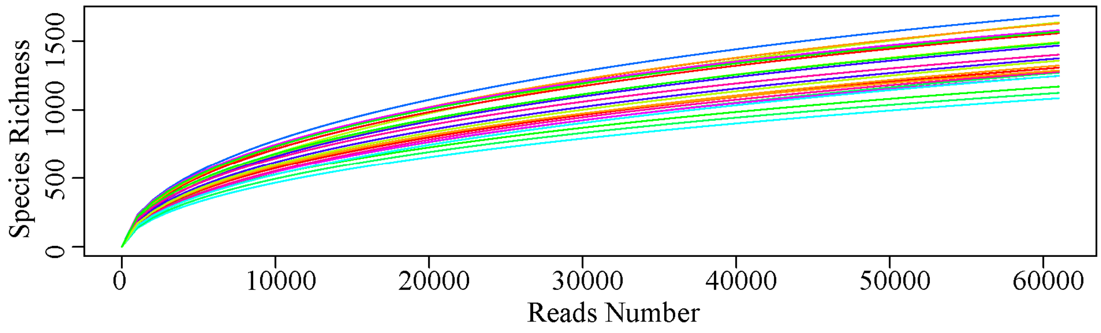

Figure S1: Rarefaction curves of the number of operational taxonomic units (OTUs) at 97% similarity boxplot for each of 24 samples.

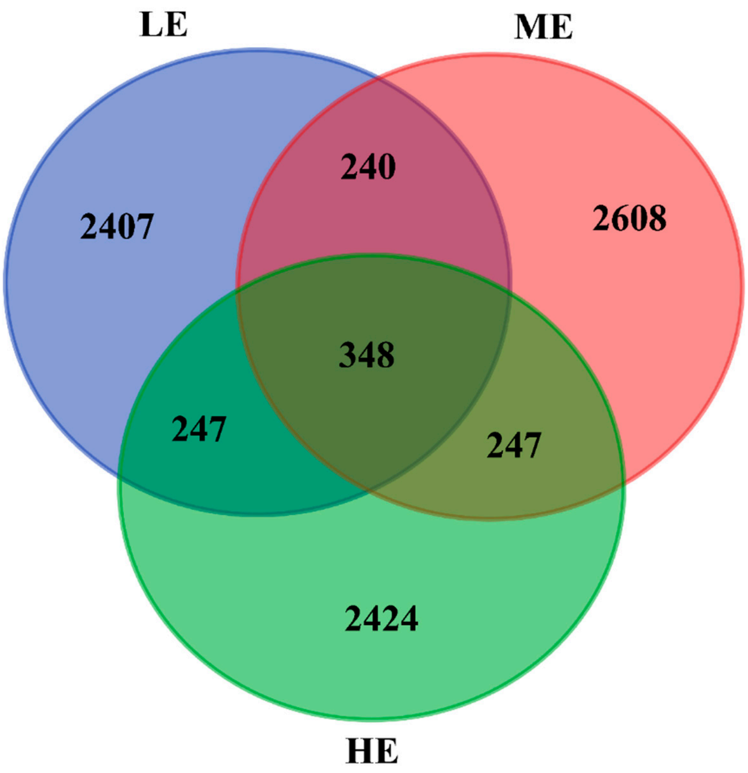

Figure S2: Venn diagrams showing endemic and shared fungal OTUs in three different elevation rhizosphere. LE: low elevation; ME: middle elevation; HE: high elevation. OTU: operational taxonomic unit.

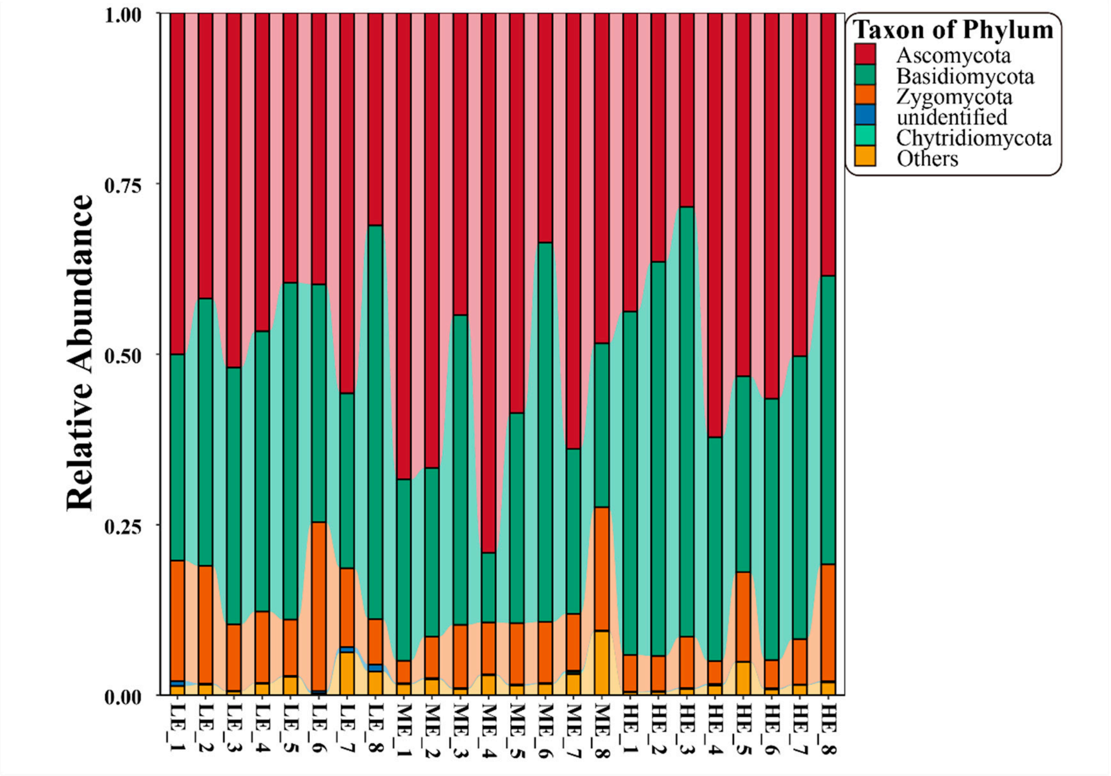

Figure S3: The relative abundance of rhizosphere fungal phyla at each sampling site.

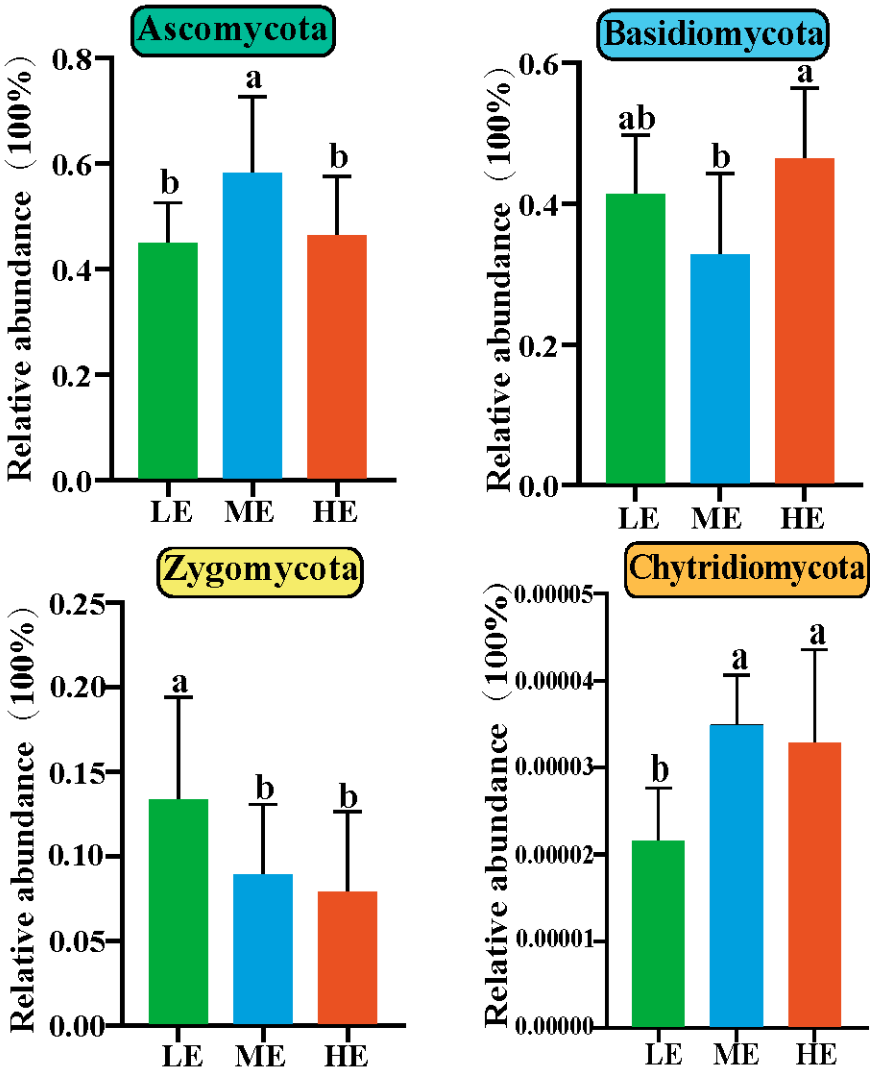

Figure S4: The differences of dominant rhizosphere fungal phyla at different elevations, letters represent significant differences from the one-way ANOVA with Duncan's comparisons ($P < 0.05$).

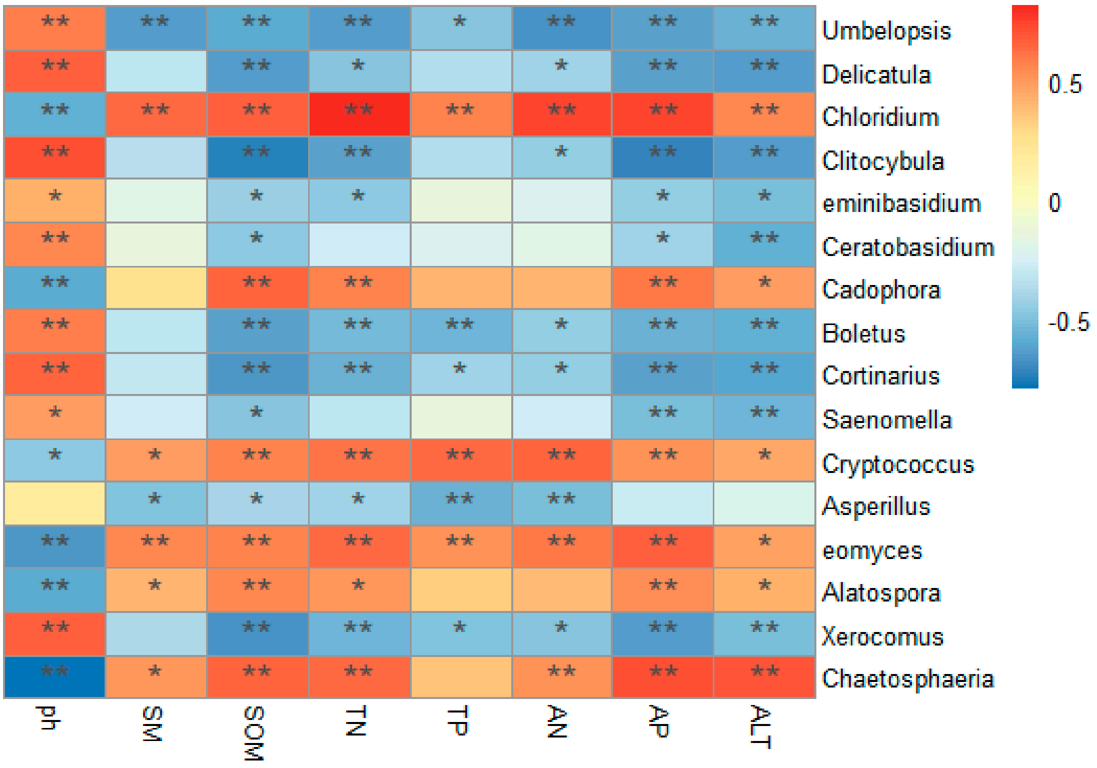

Figure S5: Spearman rank correlations between the relative abundance of dominant fungal taxa and soil properties. Relationship significant at*$P < 0.05$; **$P < 0.01$; ***$P < 0.001$.

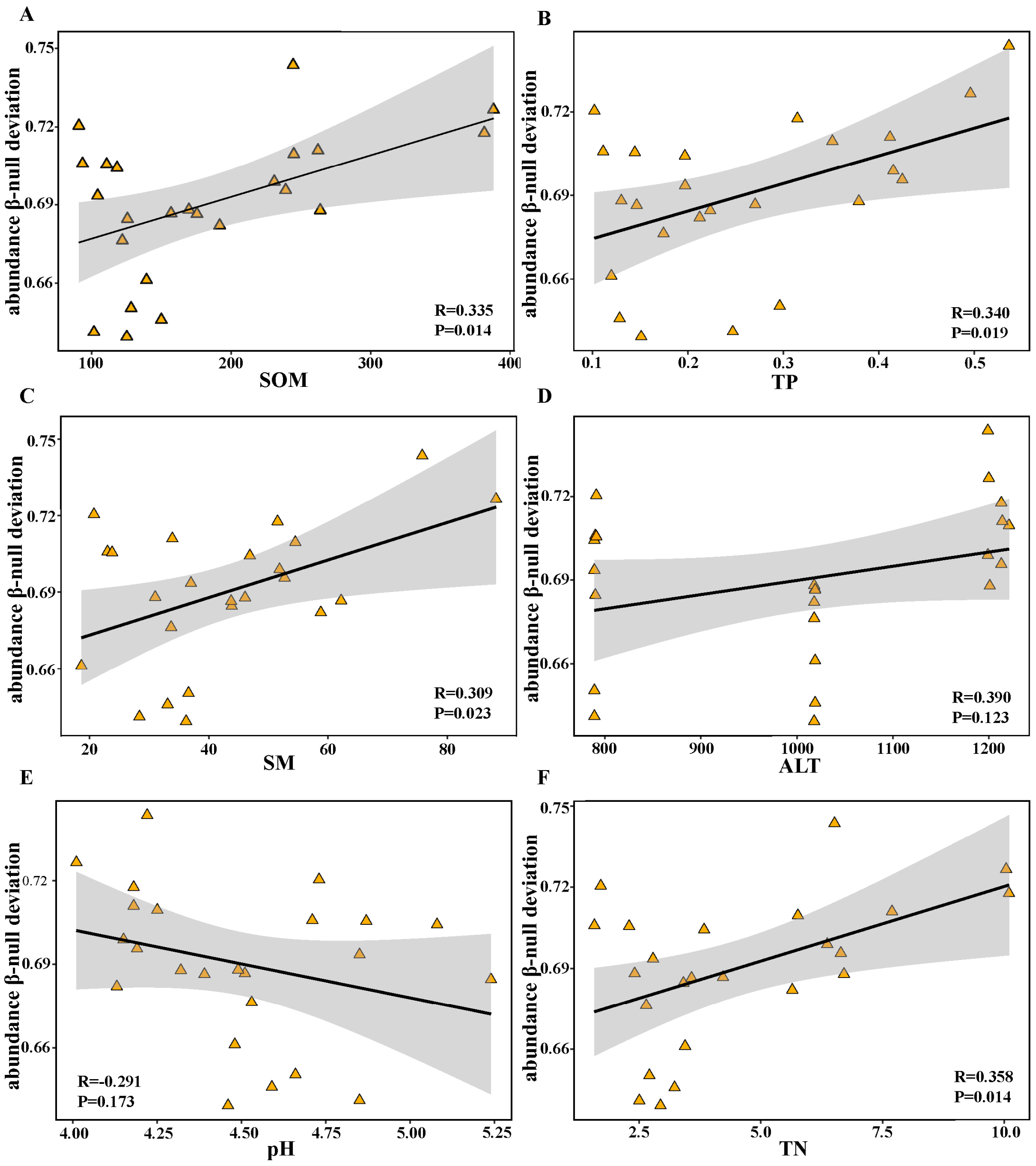

Figure S6: Linear regression analysis of soil physicochemical and β-null deviation. Grey shaded areas indicate 95% confidence intervals ($P < 0.05$).

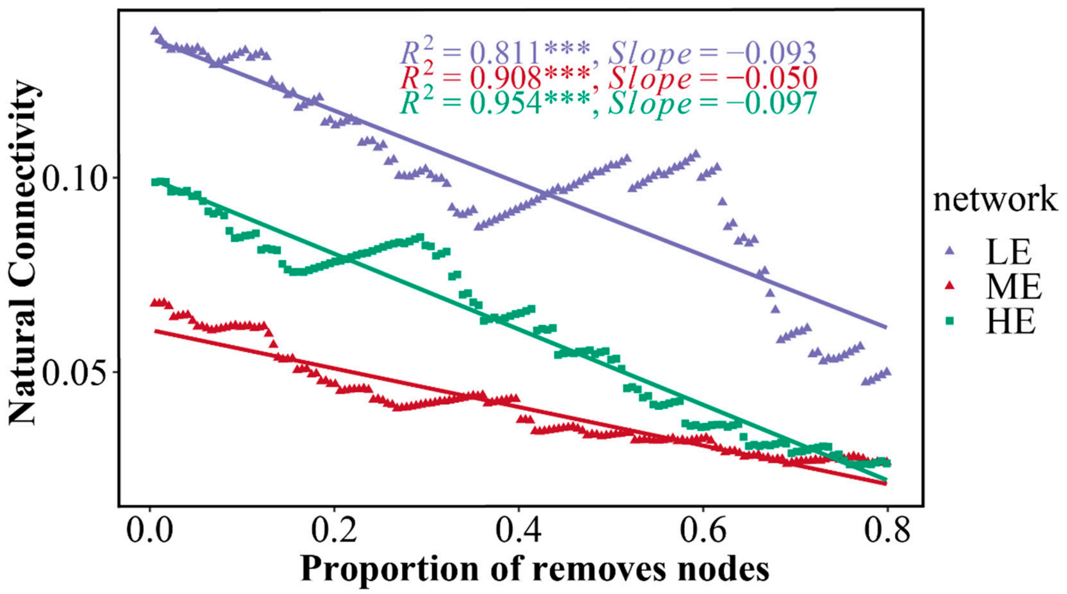

Figure S7: The stability of co-occurrence network. LE: low elevation; ME: middle elevation; HE: high elevation.
